# Amniotic Fluid Embolism Successfully Managed With Extracorporeal Life Support and Damage Control Surgery: A Case Report

**DOI:** 10.1155/crcc/3347972

**Published:** 2026-03-09

**Authors:** Taiga Nagano, Kenshin Shimono, Tomonori Hamada, Yukiko Tazaki, Yutaro Madokoro, Mitsuhito Sato, Shuhei Niiyama, Ikumi Kitazono, Shintaro Yanazume, Shinichi Togami, Hiroaki Kobayashi

**Affiliations:** ^1^ Department of Emergency and Critical Care Medicine, Kagoshima University Hospital, Kagoshima, Japan, kagoshima-u.ac.jp; ^2^ Department of Obstetrics and Gynecology, Kagoshima University Hospital, Kagoshima, Japan, kagoshima-u.ac.jp; ^3^ Department of Pathology, Field of Oncology, Kagoshima University Graduate School of Medical and Dental Sciences, Kagoshima, Japan, kagoshima-u.ac.jp

## Abstract

**Background:**

Amniotic fluid embolism can lead to severe complications, including cardiac arrest, a significant cause of maternal mortality.

**Case Presentation:**

A 36‐year‐old woman with postpartum hemorrhage was transported to our hospital. Shortly after her arrival, she experienced cardiac arrest and required immediate extracorporeal life support. A hysterectomy was performed, but persistent bleeding necessitated open abdomen management for damage control to stabilize the patient′s condition. On the second day, she was successfully weaned from extracorporeal membrane oxygenation, followed by catheter removal and complete abdominal closure. The patient was discharged with no neurological sequelae. Pathological examination of the hysterectomy specimen confirmed amniotic fluid embolism.

**Conclusion:**

Extracorporeal life support may be effective as a last resort for cardiac arrest due to amniotic fluid embolism, and damage control surgery may be beneficial for managing intractable bleeding.

## 1. Introduction

Amniotic fluid embolism (AFE) is a rare but potentially life‐threatening condition [1]. From 2010 to 2022 in Japan, AFE accounted for 11% of maternal deaths [2]. It can rapidly progress from circulatory collapse to cardiac arrest, and in some cases, conventional cardiopulmonary resuscitation (CPR) fails to achieve survival [3]. In recent years, an increasing number of cases have been reported in which venoarterial extracorporeal membrane oxygenation (ECMO), as part of extracorporeal life support (ECLS), was used to treat cardiac arrest caused by AFE [4].

AFE frequently leads to disseminated intravascular coagulation, resulting in a severe bleeding tendency [1]. Initiating ECMO can further worsen these bleeding complications, making hemorrhage control particularly difficult [5]. By contrast, in trauma care, damage control surgery (DCS) has been shown to effectively stabilize patients during initial resuscitation, enabling staged surgical interventions once the patient′s condition improves.

In this report, we describe a unique case in which ECLS and DCS—approaches traditionally used in trauma care—were successfully adapted to manage a critical case of AFE.

## 2. Case Presentation

A 36‐year‐old Japanese woman (height 152 cm, weight 56 kg, body mass index 24 kg/m^2^), Gravida 1 Para 0, was admitted to an obstetric clinic at 39 weeks of gestation in labor. She had no significant medical history, and her pregnancy was uncomplicated except for Rh‐negative blood type. Labor was initiated spontaneously but augmented with oxytocin because of inadequate uterine contractions. Because of a nonreassuring fetal status, she underwent an emergency cesarean section with an estimated blood loss of 380 mL. Following the surgery, she developed shock, and her systolic blood pressure dropped to 60 mmHg. Her condition did not improve despite the administration of approximately 1500 mL of crystalloid and 200 mL of autologous blood, as well as the use of intrauterine balloon tamponade. She was transferred to our hospital 90 min after the sudden deterioration, receiving oxygen supplementation (10 L/min), continuous fluid resuscitation, and intrauterine balloon tamponade during transport. Estimated blood loss at the referring clinic was approximately 1400 mL.

Upon arrival, her blood pressure was immeasurable, pulse rate was 120 bpm, and Glasgow Coma Scale score was E1V2M1, indicating severely altered consciousness. Suspecting severe hemorrhagic shock, we proceeded with intubation, massive blood transfusion, and resuscitative endovascular balloon occlusion of the aorta (REBOA) catheter insertion. Arterial blood gas analysis revealed the following: pH 6.8, pO_2_ 382.0 mmHg (FiO_2_ 1.0), pCO_2_ 25.2 mmHg, HCO_3_^−^ 4.4 mmol/L, hemoglobin 3.3 g/dL, lactate 20.0 mmol/L, and intra‐abdominal hemorrhage confirmed by ultrasonography. Her pulse soon deteriorated to pulseless electrical activity, necessitating CPR. Despite the relatively limited blood loss (estimated at 1400 mL at the referring clinic), the patient rapidly developed profound acidosis (pH 6.8, lactate 20.0 mmol/L) and progressed to cardiac arrest within minutes of arrival. This clinical course raised suspicion of AFE in addition to hemorrhagic shock as the underlying cause. Because REBOA catheter insertion had only just begun, we transitioned to ECMO catheter insertion at the same site to address circulatory failure. Blood transfusion was initiated, and ECMO was commenced 18 min after the onset of cardiac arrest. The ECMO circuit was primed with heparinized saline (2000 units), and the pump speed was set at 1800 rpm, maintaining flow of approximately 2.0–2.5 L/min. Body temperature was maintained at 36.5°C using a heat exchanger to prevent hypothermia‐induced coagulopathy. Given the ongoing bleeding, no additional systemic heparin was administered after ECMO initiation, and anticoagulation‐free management was continued throughout the ECMO course. Following ECMO initiation, the patient showed eye‐opening–like movements in response to verbal stimulation and was transferred to the operating room for hemorrhage control. Surgery revealed abdominal bleeding; despite efforts to control it, uterine bleeding continued, necessitating a hysterectomy (Figure 1a). After the hysterectomy, persistent oozing and venous bleeding from multiple abdominal sites necessitated gauze packing and an open abdomen under DCS (Figure 1b). After transfer to the intensive care unit, the patient received massive transfusion—including whole blood—and fibrinogen concentrate. Intraoperative blood loss was estimated at 5000 mL. Following ICU admission, ongoing bleeding from the open abdomen continued initially, with an additional estimated 5000 mL of blood loss before hemostasis was gradually achieved through correction of coagulopathy. Adjunctive therapies included tranexamic acid for its antifibrinolytic effect and continuous renal replacement therapy to correct severe metabolic acidosis. These interventions, combined with DCS, led to improvement of coagulopathy and hemodynamic stability. On Day 2, following further improvement, abdominal closure and removal of the ECMO catheter were performed (Figure 1c).

Figure 1Clinical management of amniotic fluid embolism leading to cardiac arrest in a 36‐year‐old woman. (a) Initial surgical intervention showing the noncontracting uterus with continuous bleeding, necessitating a hysterectomy. (b) Posthysterectomy venous bleeding and oozing were managed with gauze packing and open abdomen techniques as part of the damage control strategy. (c) Following open abdomen management, improvement of the coagulopathy led to reduced bleeding, allowing for subsequent complete abdominal closure and removal of the extracorporeal membrane oxygenation catheter the next day.

**Figure:**
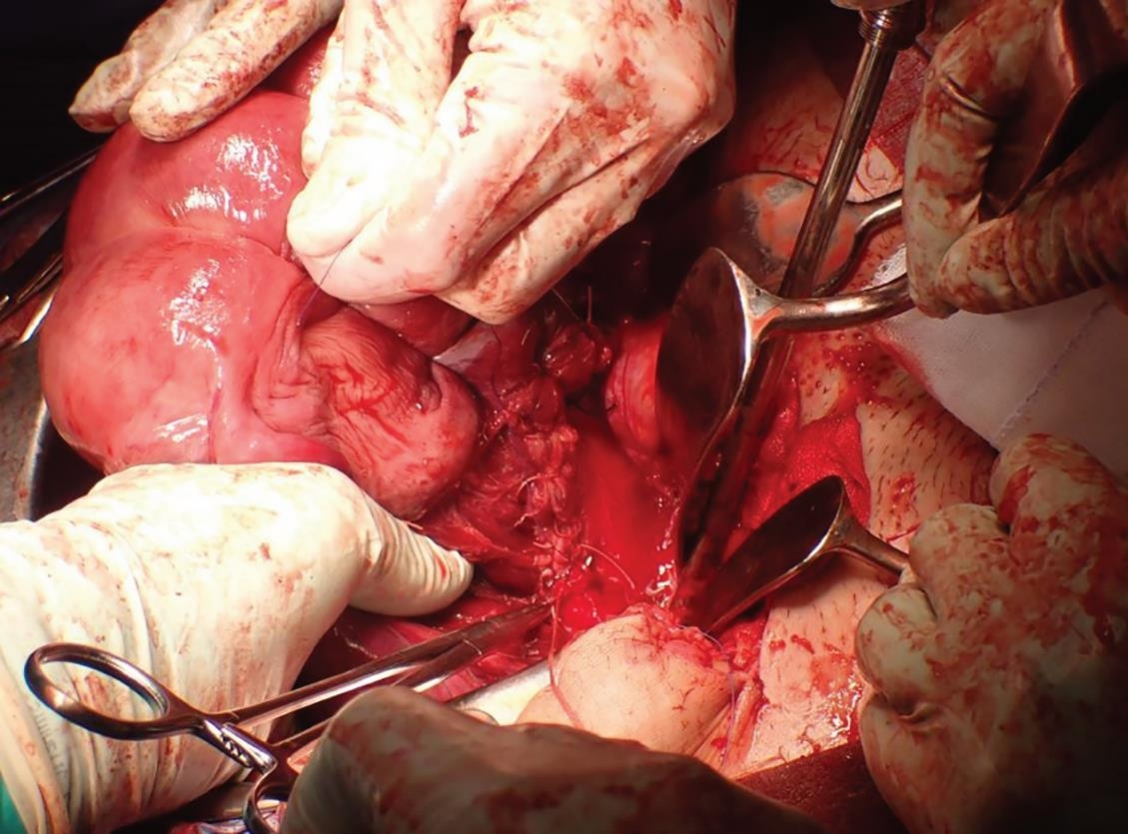
(a)

**Figure:**
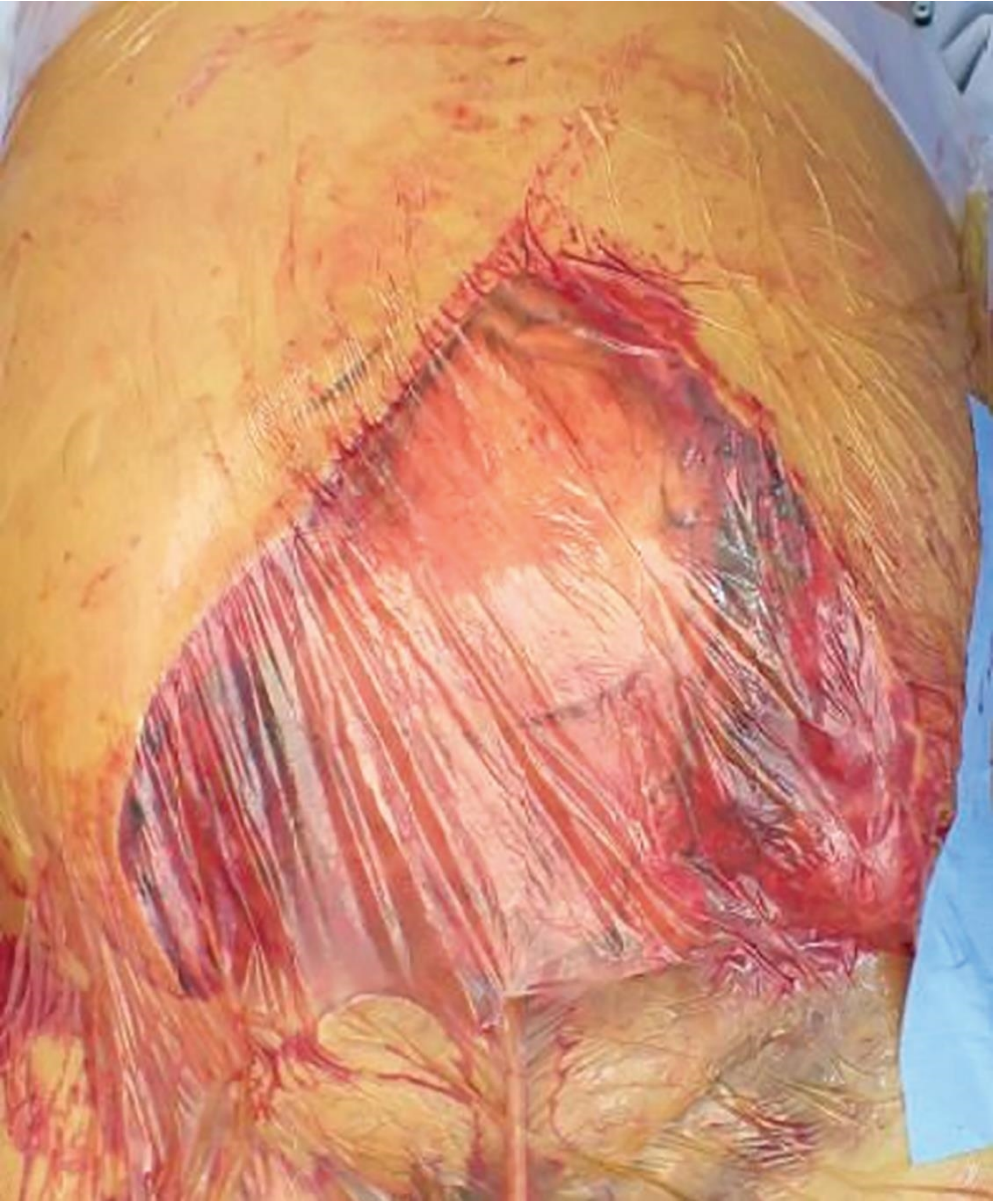
(b)

**Figure:**
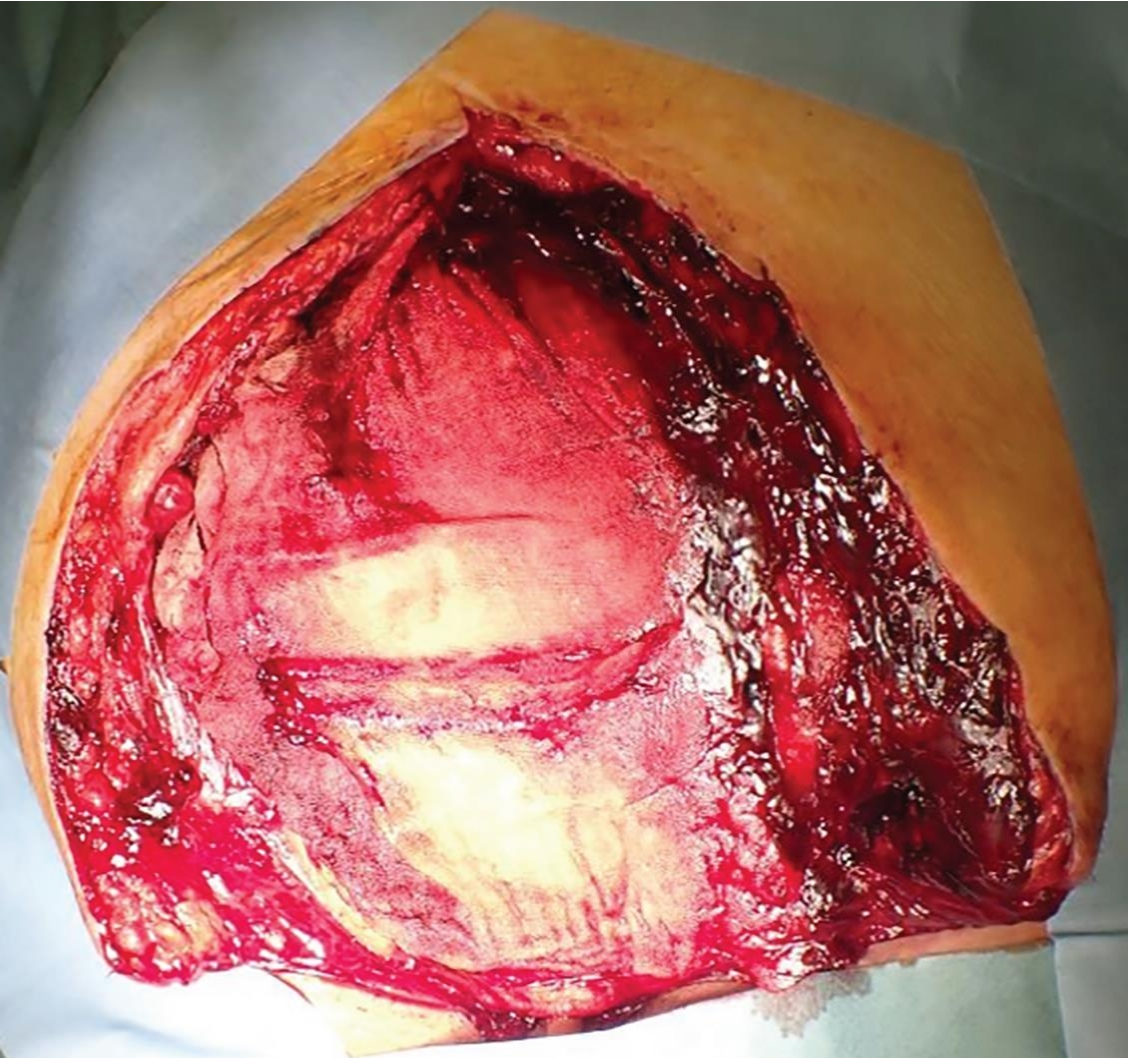
(c)

During her hospital stay, the patient received 90 units of red blood cells, 86 units of fresh frozen plasma, 60 units of platelet concentrate, and 5800 mL of whole blood. She had several recognized risk factors for AFE, including advanced maternal age, oxytocin use, and cesarean delivery.

Histological examination of the resected uterus confirmed the diagnosis of AFE (Figure 2). Macroscopically, a cervical laceration was observed, extending into the deep muscular layer and accompanied by hemorrhage (Figure 2a,b). Microscopically, multiple dilated blood vessels within the cervical wall contained lamellar keratinized material consistent with fetal squamous cells, along with numerous inflammatory cells including lymphocytes and neutrophils (Figure 2c,d). Scattered thrombi were also present (Figure 2e). Immunohistochemical staining with AE1/AE3 showed positive reactivity in the keratinized material, confirming its epithelial origin (Figure 2f). These pathological findings supported a diagnosis of classic AFE rather than iatrogenic thromboembolism.

Figure 2Histopathological evidence of amniotic fluid embolism. Macroscopic findings. (a) Resected uterus with cervical laceration. (b) Cross section of the laceration site (red box indicates area shown in (c)). Microscopic findings. (c) Low‐power view of the cervical wall showing multiple dilated blood vessels (yellow arrows) containing intraluminal material (hematoxylin–eosin staining, loupe view). (d) Magnified view (×20) of a dilated vessel (yellow box from (c)). (e) High‐power view (×200) showing the vessel lumen containing red blood cells, inflammatory cells (lymphocytes and neutrophils), and characteristic lamellar keratinized material (yellow box from (d)). (f) AE1/AE3 immunohistochemistry (×200) showing positive staining of the keratinized material, confirming its fetal squamous cell origin. These findings are diagnostic of amniotic fluid embolism.

**Figure:**
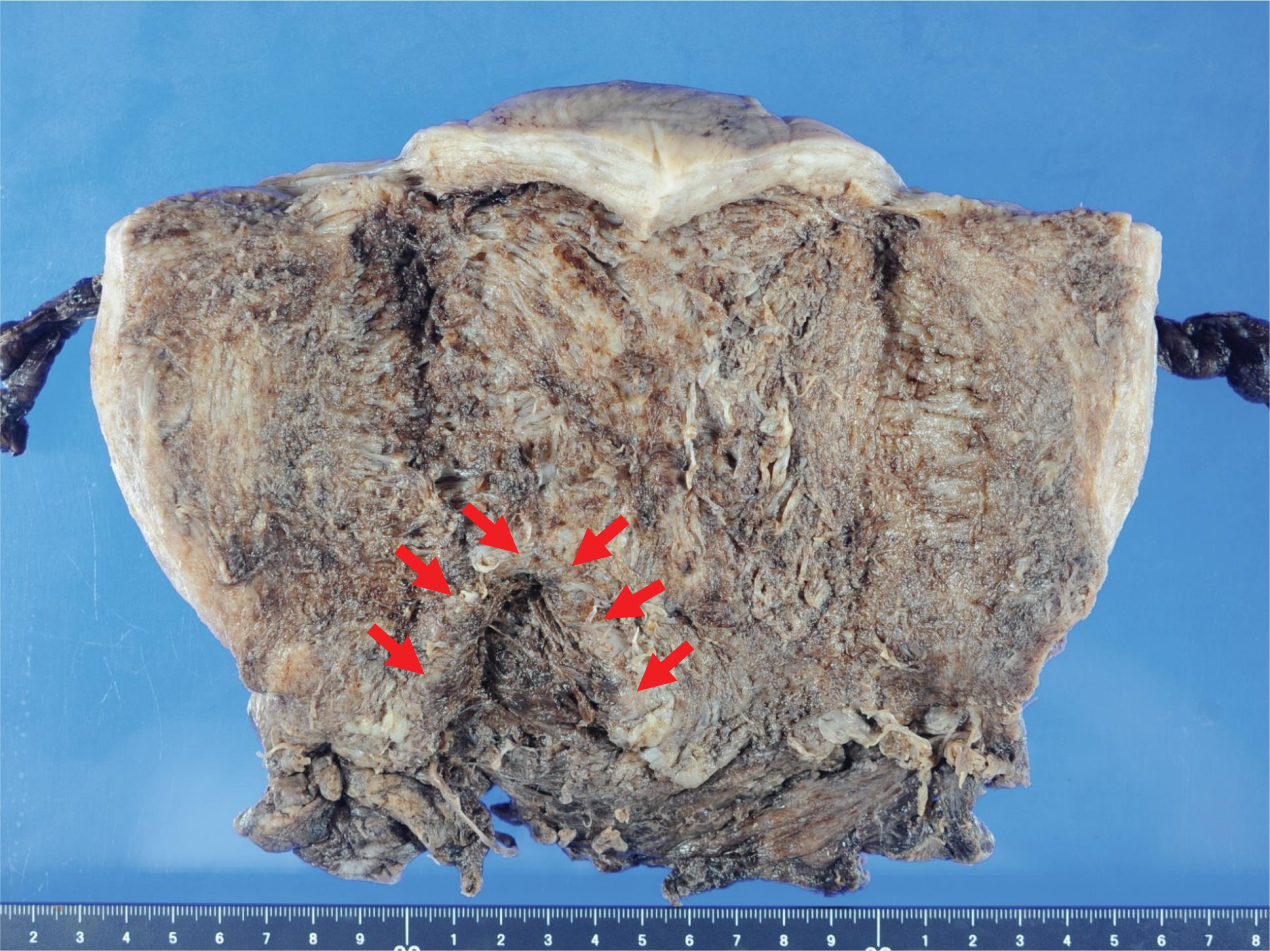
(a)

**Figure:**
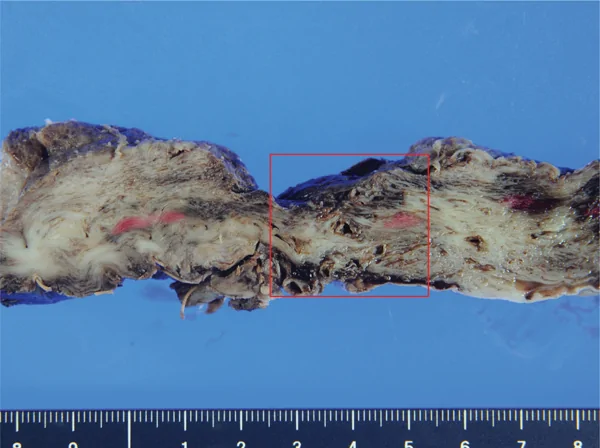
(b)

**Figure:**
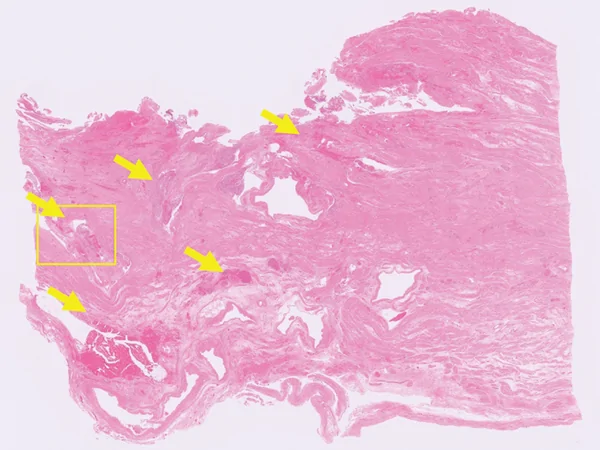
(c)

**Figure:**
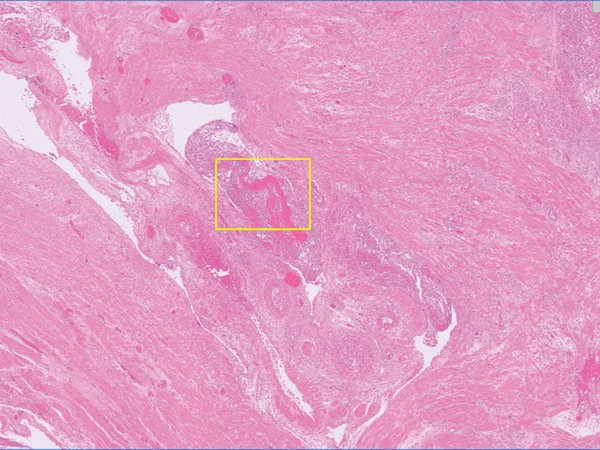
(d)

**Figure:**
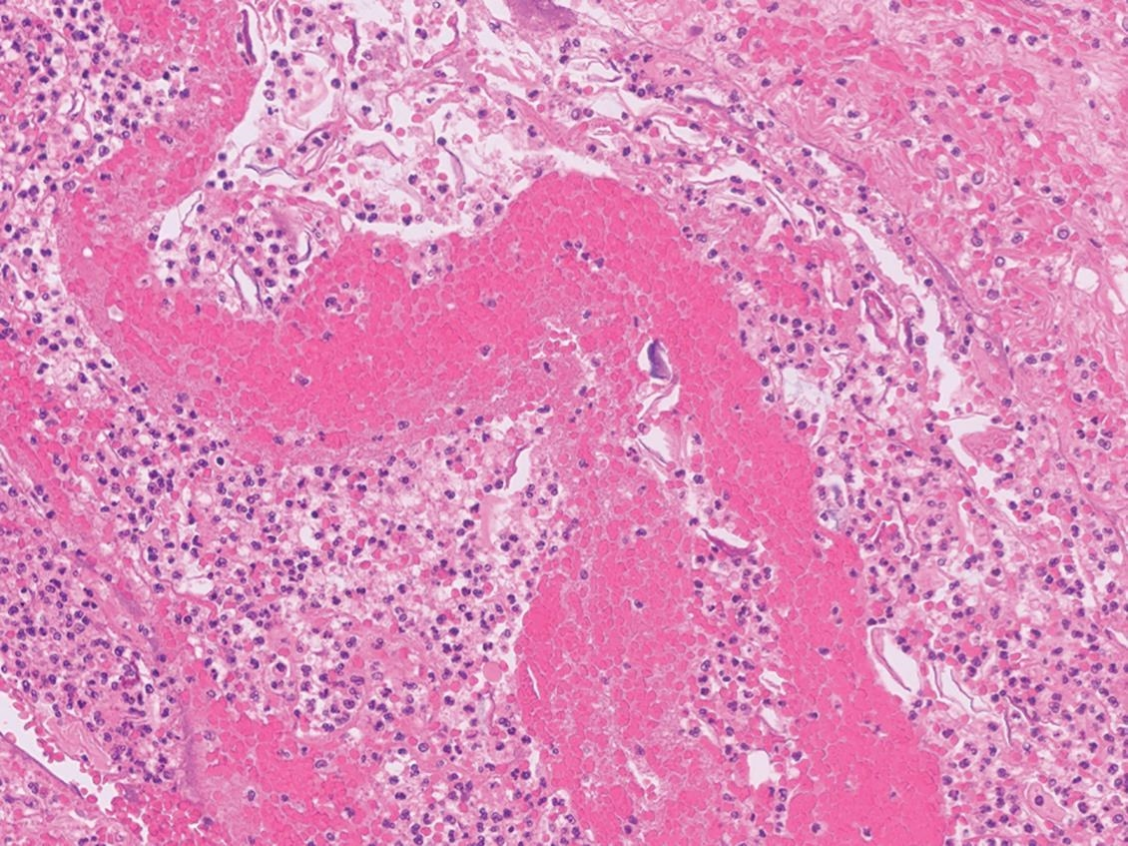
(e)

**Figure:**
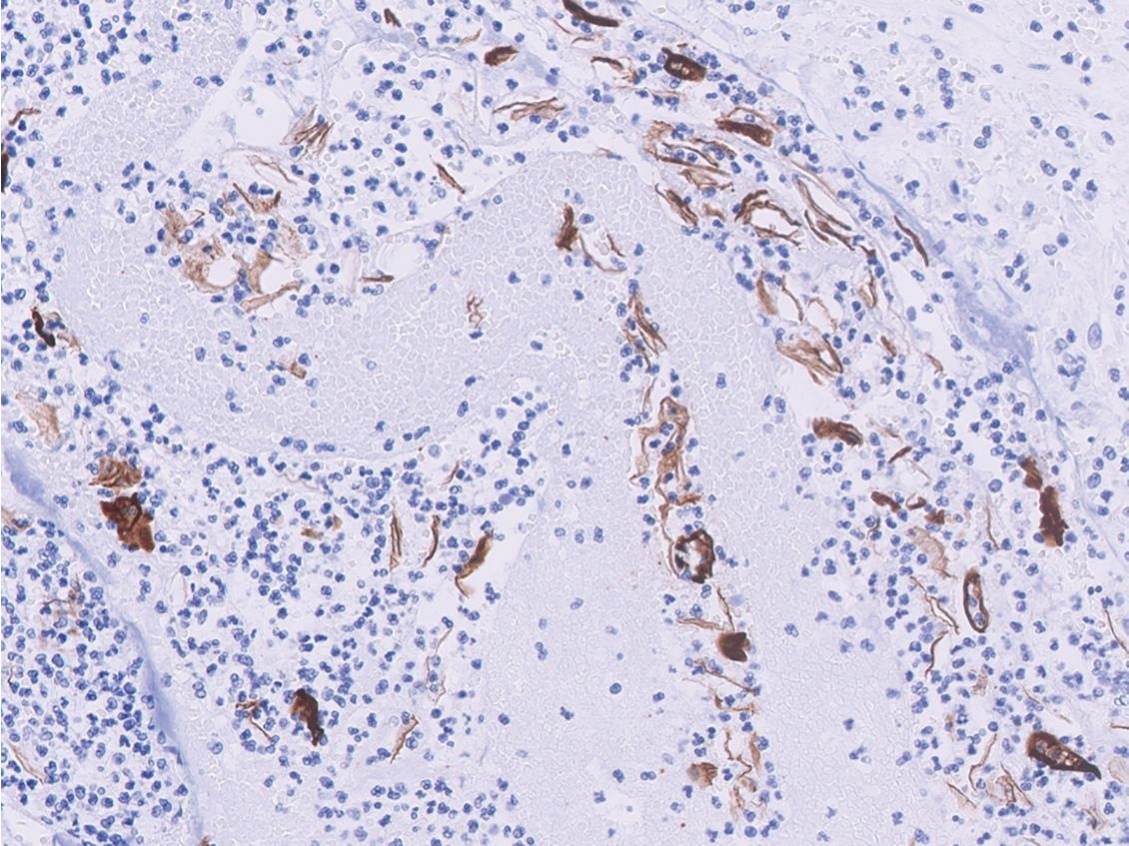
(f)

The patient subsequently developed paralytic ileus, arteriovenous thrombosis, and hemolytic anemia, all of which resolved, allowing for discharge on Day 42 without neurological sequelae. Tables 1, 2, and 3 summarize the management, timeline of interventions, and clinical outcomes in this case of AFE complicated by cardiopulmonary arrest.

**Table 1:** Management and outcome of cardiopulmonary arrest due to amniotic fluid embolism in a 36‐year‐old woman.

Category	Information	Details
Patient situation	Age (years)	36
	Gravidity/parity	G1P0
	Gestational age (week)	39
	Delivery	Caesarian section
	Blood type	A−
	Timing of sudden change	After caesarian section
	Etiology	PPH + AFE

Mechanical support	ECMO initiation	During CPR
	ECMO type	Venoarterial
	Heparin dose at ECMO priming	2000 units
	Low flow time (min)	18
	ECMO duration (h)	6

Rescue status	Total blood transfusion	RBC 90 units, FFP 86 units, PC 60 units, whole blood 30 units
	Other rescue treatment	TAH, open abdomen management

Prognosis	Mother outcome	Survived
	Fetus outcome	Survived

Abbreviations: AFE, amniotic fluid embolism; CPR, cardiopulmonary resuscitation; ECMO, extracorporeal membrane oxygenation; FFP, fresh frozen plasma; PC, platelet concentrate; PPH, postpartum hemorrhage; RBCs, red blood cells; TAH, total abdominal hysterectomy; U, Japanese units.

**Table 2:** Timeline of interventions after hospital admission.

Time after arrival (hour:min)	Event
0:00	Admission to hospital
0:02	Transabdominal ultrasonography performed
0:05	Start of REBOA insertion
0:07	Initiation of CPR
0:08	Intubation
0:08	Discontinuation of REBOA, start of ECMO catheter insertion
0:17	Start of blood transfusion
0:25	Activation of ECMO pump
0:56	Transfer to operating room
1:09	Commencement of surgery
2:19	Hysterectomy performed
4:07	Gauze packing and open abdomen management
4:18	Transfer to ICU
5:49	Whole blood transfusion administered
6:34	Deactivation of ECMO pump

**Table 3:** Postadmission outcomes and complications.

Day after admission (day)	Event
2	Abdominal closure completed, ECMO catheter removed
4	Extubation
5	Discharge from ICU
9	Development of arteriovenous thrombosis, onset of paralytic ileus
24	Development of hemolytic anemia
42	Discharge from hospital

Abbreviations: CPR, cardiopulmonary resuscitation; ECMO, extracorporeal membrane oxygenation; ICU, intensive care unit; REBOA, resuscitative balloon occlusion of the aorta.

## 3. Discussion

This case offers valuable insights into the management of AFE. First, it demonstrates that ECLS can be a viable resuscitation option even in cases of AFE. Second, damage control strategies—such as the open abdomen technique—may be essential for stabilizing critically compromised patients during surgery.

ECLS has been shown to improve survival rates, even in peripartum patients. Survival rates for peripartum cardiac arrest have been reported to improve with ECPR, rising from 59% to 87%, and for AFE, from 52% to 85% [6]. Another key advantage of ECLS during cardiopulmonary arrest is its ability to maintain cerebral blood flow [7], rapidly increasing cerebral oxygen saturation compared with conventional CPR [8]. Traditional CPR achieves only 20%–30% of normal cardiac output [9]. In this case, the patient had a favorable neurological outcome despite 18 min of cardiac arrest, underscoring the potential of ECLS to preserve brain function in acute settings.

The typical presentation of AFE includes a triad of sudden hypoxia and hypotension, often followed by coagulopathy [1]. These conditions can progress to hypothermia and acidosis, completing the “lethal triad” that includes coagulopathy and may ultimately lead to death [10]. However, ECMO helps regulate body temperature through a heat exchanger, maintaining it at 36°C and thereby preventing hypothermia. Guidelines emphasize the importance of managing the patient′s condition after resuscitation, particularly in cases of hypoxic–ischemic encephalopathy [11]. ECMO offers both respiratory and circulatory support to improve acidosis, potentially addressing two components of the lethal triad: hypothermia and acidosis.

Nevertheless, ECLS can be complicated by hemorrhage in 26%–35% of trauma patients [12, 13]. However, it has been safely used in cases of traumatic brain injury without anticoagulation. This has led to strategies such as “heparin‐free” ECLS and adjustments to clotting time based on bleeding risk [14]. Even in the presence of severe disseminated intravascular coagulation and active bleeding, the use of venoarterial ECMO in peripartum patients has been shown to be beneficial [5]. In this case, the ECMO circuit was primed with heparinized saline, but no systemic heparin was administered thereafter because of active bleeding. This heparin‐free strategy during ECMO support proved safe and effective in managing severe coagulopathy.

DCS can be crucial for managing uncontrollable bleeding, particularly in postpartum cases [15]. Originally developed for trauma care [16], DCS has proven effective in obstetrics, helping to stabilize patients who are not suitable for prolonged surgery [15]. It enables staged surgical interventions following initial resuscitation, which is especially important in cases involving coagulopathy. In this case, gauze packing and open abdomen techniques were employed as part of the DCS approach.

## 4. Conclusion

ECLS may serve as a last‐resort option for patients experiencing cardiac arrest due to AFE. Additionally, DCS appears to be an effective strategy for managing coagulopathy that is difficult to control.

## Funding

No funding was received for this manuscript.

## Ethics Statement

The patient provided written consent to submit this case report for publication.

## Conflicts of Interest

The authors declare no conflicts of interest.

## Data Availability

Data will be made available on request.
